# Heart Morphogenesis Requires Smyd1b for Proper Incorporation of the Second Heart Field in Zebrafish

**DOI:** 10.3390/genes16010052

**Published:** 2025-01-04

**Authors:** Kendal Prill, Pamela Windsor Reid, Dave Pilgrim

**Affiliations:** 1 Department of Biological Sciences, University of Alberta, Edmonton, AB T6G 2E9, Canada; kprill@uoguelph.ca (K.P.); reidp22@macewan.ca (P.W.R.); 2 Department of Molecular and Cellular Biology, University of Guelph, Guelph, ON N1G 1Y2, Canada; 3 Department of Biological Science, MacEwan University, Edmonton, AB T5J 4S2, Canada

**Keywords:** zebrafish, heart development, second heart field, Smyd1, smyd1b, cardiac sarcomere, extracellular matrix, still heart mutant

## Abstract

**Background/Objectives:** Abnormal development of the second heart field significantly contributes to congenital heart defects, often caused by disruptions in tightly regulated molecular pathways. *Smyd1*, a gene encoding a protein with SET and MYND domains, is essential for heart and skeletal muscle development. Mutations in SMYD1 result in severe cardiac malformations and misregulation of *Hand2* expression in mammals. This study examines the role of Smyd1b in zebrafish cardiac morphogenesis to elucidate its function and the mechanisms underlying congenital heart defects. **Methods:** Smyd1b (*still heart*) mutant embryos were analyzed for cardiac defects, and changes in gene expression related to heart development using live imaging, in situ hybridization, quantitative PCR and immunofluorescent comparisons and analysis. **Results:** Smyd1b mutants displayed severe cardiac defects, including failure to loop, severe edema, and an expansion of cardiac jelly linked to increased *has2* expression. Additionally, the expression of key cardiac transcription factors, such as *gata4*, *gata5*, and *nkx2.5*, was notably reduced, indicating disrupted transcriptional regulation. The migration of cardiac progenitors was impaired and the absence of Islet-1-positive cells in the mutant hearts suggests a failed contribution of SHF progenitor cells. **Conclusions:** These findings underscore the essential role of Smyd1b in regulating cardiac morphogenesis and the development of the second heart field. This study highlights the potential of Smyd1b as a key factor in understanding the genetic and molecular mechanisms underlying congenital heart defects and cardiac development.

## 1. Introduction

Congenital heart defects affect approximately 1% of live births, with a higher incidence of preterm mortalities [1,2,3]. The mature heart is formed from two distinct progenitor cell lineages of the splanchnic mesoderm, the first heart field (FHF) and the second heart field (SHF) [4,5]. FHF-derived cells primarily contribute to the formation of the left ventricle and part of the atria, while SHF progenitors give rise to the right ventricle, atria, atrial and ventricular septal structures, and the outflow tract [5,6,7]. Defects in the SHF and its cardiac progenitors result in a wide spectrum of cardiac malformations due to the intricate processes involved in heart morphogenesis that are regulated by complex and intersecting signaling pathways [8,9].

The heart tube is constructed through the fusion of bilateral precardiac regions, which first form into the cardiac crescent at the embryonic ventral midline. The population of SHF progenitor cells are medially separated from the developing heart tube (FHF) by the breakdown of dorsal mesocardium but remains contiguous at the arterial and venous poles [5,10]. The second heart field progenitor cells have delayed differentiation and continued proliferation, which allows for continued contribution to the heart during elongation and morphogenesis [11]. SHF cell proliferation and differentiation programs are controlled by signals from the surrounding pharyngeal region [11,12,13,14]. Proliferative and undifferentiated SHF cells express transcription factors *Fgf10, Fgf8, tbx1*, and *isl1* [14,15,16]. Upon cardiac specification and SHF cell migration to the developing heart, these cells downregulate factors of the proliferative program and begin to express genes of the cardiac program such as *mef2c*, *nkx2.5*, *gata4*, and *tbx5* [14,17,18,19]. Each chamber and tissue of the heart has its own transcriptional program [5,14] and our understanding of these signaling pathways is incomplete largely due to the embryonic lethality of SHF mutations in avian and mammalian models [6,18,20,21].

Mutants of *smyd1*, a gene encoding a protein with SET and MYND domains, have nonfunctional and underdeveloped hearts [22,23,24]. Variants in human Smyd1 are associated with cardiomyopathies and cardiac failure, which suggests Smyd1 is a critical factor required for normal heart development and function [25,26,27,28]. In mice, Smyd1 is proposed to regulate *hand2* expression, which is important for proper looping of the heart tube [22]. Despite protein domains required for histone methyltransferase activity and protein–protein interactions, there is evidence that Smyd1 functions as a myosin chaperone during skeletal muscle development and that the heart defects seen in mice and zebrafish could be due to defective cardiac sarcomere formation and a lack of contraction needed for looping [24,29,30,31,32,33]. In the zebrafish *smyd1b* mutant, *still heart*, a nonfunctional heart forms but the atrium and ventricle are abnormally shaped and sized [24].

Zebrafish provide a powerful system for studying embryonic heart development and lethal mutations, as a functional heart is not required for development to proceed for up to five days post-fertilization. This advantage, combined with the rapid development, short generation time, and transparent embryos of zebrafish, allows for the detailed resolution of cellular differences in a living system [34,35]. Additionally, heart development and the signaling pathways are conserved across vertebrates, making discoveries in zebrafish translatable to higher-order vertebrates. Zebrafish go through similar stages of heart development and morphogenesis as birds and mammals but create a two-chambered heart instead of four [36]. The zebrafish second heart field contributes cells to both chambers and is primarily responsible for the formation of the inflow and outflow tracts, making any defects in SHF development detrimental to heart formation, morphogenesis and function [37,38,39,40].

Given the conserved role of Smyd1 in heart development across vertebrates, there are three potential roles for Smyd1 during embryogenesis. First, as demonstrated in mice, Smyd1 is essential for regulating the transcription of key factors involved in heart morphogenesis [22,23,41]. Second, evidence from zebrafish highlights Smyd1b’s role as a myosin chaperone during skeletal muscle development, suggesting a similar function in cardiac sarcomere assembly and heart contraction during morphogenesis [24,30,32,42]. Lastly, with its SET, MYND, and myosin-binding domains, Smyd1b may function as both a histone methyltransferase and a myosin chaperone during heart development, potentially influencing chromatin remodeling and sarcomere organization simultaneously.

Previous work has demonstrated that *smyd1b* null mutants lack sarcomeres and myofibril organization in their cardiac tissue [30], which supports the hypothesis that Smyd1b is required for normal cardiac sarcomere assembly. We therefore explored the possibility and role of Smyd1b as a factor involved in regulating cardiac development, by studying heart morphogenesis in the *smyd1b* mutant *still heart* [24,31,43]. We compared the expression of key transcription factors in heart development between *still heart* and wild-type embryos to assess whether Smyd1b plays a regulatory role in gene expression.

We found that Smyd1b is essential for proper heart morphogenesis, and specifically, Smyd1b is required for the proper expression of key cardiac transcription factors, including *gata4*, *nkx2.5*, *tbx5a/b*, and *hand2*. Consistent with findings in *smyd1* mutant mice, *hand2* expression was significantly reduced in *still heart* mutants, with the abnormal localization of *hand2*-expressing cells observed during heart development. Moreover, the *still heart* mutants exhibited a notable absence of Isl1-positive cells in the heart at 36 and 48 hpf, indicating that Smyd1b plays a critical role in the incorporation of second heart field cells into the developing heart. These findings highlight the importance of Smyd1b in orchestrating transcriptional regulation and cell migration during cardiac morphogenesis. 

## 2. Materials and Methods

### 2.1. Ethics Statement

All procedures/methods were performed following the guidelines stated by the Canadian Council for Animal Care and the protocols approved by the Animal Care and Use Committee of the University of Alberta (Animal Use permit AUP#00038).

### 2.2. Zebrafish Husbandry, Strain Maintenance, and Animal Use

All adult zebrafish were bred and maintained according to standard procedures [44], and kept at 28 °C on a day/night cycle of 14 h light/10 h dark. Adults were housed in a cycled-water aquatic facility and fed twice daily with brine shrimp. Embryos were raised at 28 °C in standard embryo medium for up to 3 days prior to fixation [44].

The *still heart^tm123a^* (*sth*) zebrafish mutant was identified in the Tubingen (TB) genetic background from an ENU mutagenesis screen [45] and contains a truncation mutation in the *smyd1b* gene, a G to A transition that introduces a premature stop codon between exon 1 and 2 [24]. The *still heart* strain was maintained as a heterozygote in the AB genetic background, then crossed to WIK (Wild Indian Karyotype) for alternating generations.

Sample sizes were determined for each experiment to accommodate the experimental design, account for natural variability in development, and provide statistical power with careful consideration of reducing animal use. For each experiment, embryos collected from each genotype/stage were selected at random from a plate of embryos. There were no criteria for exclusion and no excluded animals. Wild-type and *still heart* embryos were treated and compared the same across all experiments to control for confounders.

### 2.3. In Situ Hybridization and Immunostaining

In situ hybridization was performed on whole-mount zebrafish embryos preserved overnight at 4 °C in 4% paraformaldehyde (PFA), as previously published [46]. The probe used was zebrafish *hand2* (F 5′ CCATGAGTTTAGTTGGAGGGTTTCC 3′; R 5′ TCGGTTTGATTCATAATGGGACAAG 3′). Each in situ hybridization was carried out 3 times comparing wild-type and *still heart* broods with 15 embryos per in situ; a total of 270 embryos were compared.

For immunostaining of embryo hearts, embryos were fixed overnight at 4 °C and incubated in PBST for 1 day at 4 °C. Unlike skeletal muscle immunostaining, embryos for heart imaging were not permeabilized in acetone. Embryos were washed 8 × 15 min in PBST, incubated for 1 h in 5% goat serum + PBST and then incubated in ISL1 primary antibody (ISL1, 40.2D6, DSHB; 10% primary antibody, 5% goat serum + PBST) overnight at 4 °C. The following day, embryos were washed 4 × 15 min in PBST and then incubated overnight in secondary antibody (5% secondary, 1% phalloidin + PBST; Goat anti-mouse IgG (H+L) Alexa fluor^®^ 488, Thermo Fisher Scientific, Alexa Fluor^®^ 568 Phalloidin, Thermo Fisher Scientific, South Logan, UT, USA) at 4 °C. After secondary incubation, embryos were washed 3 × 15 min in PBST and mounted for fluorescent imaging. Antibody staining was performed 3 times with embryos from 3 separate broods and 20 embryos per genotype/stage. A total of 240 embryos were used in this analysis.

The embryos were wet-mounted for whole-mount confocal imaging using a Nikon Eclipse 80i confocal microscope. Images were subsequently processed using NIS Elements version C4 software.

### 2.4. RNA Extractions and Quantitative PCR

Embryos were staged, dechorionated, had the ZEM removed, and flash-frozen in liquid nitrogen. Frozen embryo collections were homogenized for 3 min on ice in 500 µL TRIzol (Invitrogen, Carlsbad, CA, USA) using an RNAse-free pestle. Following homogenization, 250 µL chloroform was added, and tubes were inverted 6 times and centrifuged for 10 min at 13,000 RPM at 4 °C. The colorless aqueous phase was transferred to a new microfuge tube, an equal volume of chloroform was added and the tube was inverted 6 times. This new mixture was centrifuged again for 10 min at 13,000 RPM at 4 °C. Sample aqueous phases were transferred to a new microfuge tube and 2X aqueous volume of ice-cold 95% ethanol was added. Samples were inverted and incubated on ice for 30 min to precipitate. After precipitation, samples were centrifuged for 10 min at 13,000 RPM at 4 °C to collect a pellet. Supernatant was aspirated and DNA/RNA pellets were DNAse I-treated (Ambion, Austin, TX, USA; 10 μL buffer, 1 μL DNAse, 89 μL DEPC- treated distilled water) for 30 min at 37 °C. Following DNAse treatment, RNA samples were measured for purity and concentration. RNA samples were diluted to 100 ng/µL and stored at −80 °C.

cDNA was synthesized from 1 µg of RNA from each sample using qScript cDNA SuperMix (Quanta Biosciences, Beverly Hills, CA, USA) following the manufacturer’s protocol. Quantitative PCR was performed using 5 ng cDNA, 1.6 µM primer mix, and 2X SYBR mix (Molecular Biology Services Unit, University of Alberta). Primer sequences for qPCR are available in Table 1. For qPCR, we analyzed three biological replicates, with each replicate consisting of 30 embryos (90 embryos total for each genotype and stage). The relative fold change in the expression of cardiac genes in *still heart* mutants was analyzed using the 2^-ΔΔ*Ct*^ method, normalizing our wild-type samples to 1 with *ef1a* as our reference gene. A Shapiro–Wilk test was performed to assess normality, followed by parametric *t*-tests and a non-parametric Mann–Whitney U test (for *nppa* expression) to determine statistical significance. Error bars represent standard deviation of all 3 biological samples. In figures, asterisks represent the level of statistical significance: * = *p* ≤ 0.05, ** = *p* ≤ 0.01, *** = *p* ≤ 0.001.

## 3. Results

### 3.1. Smyd1b Mutant Hearts Fail to Complete Morphogenesis

**Figure 1:**
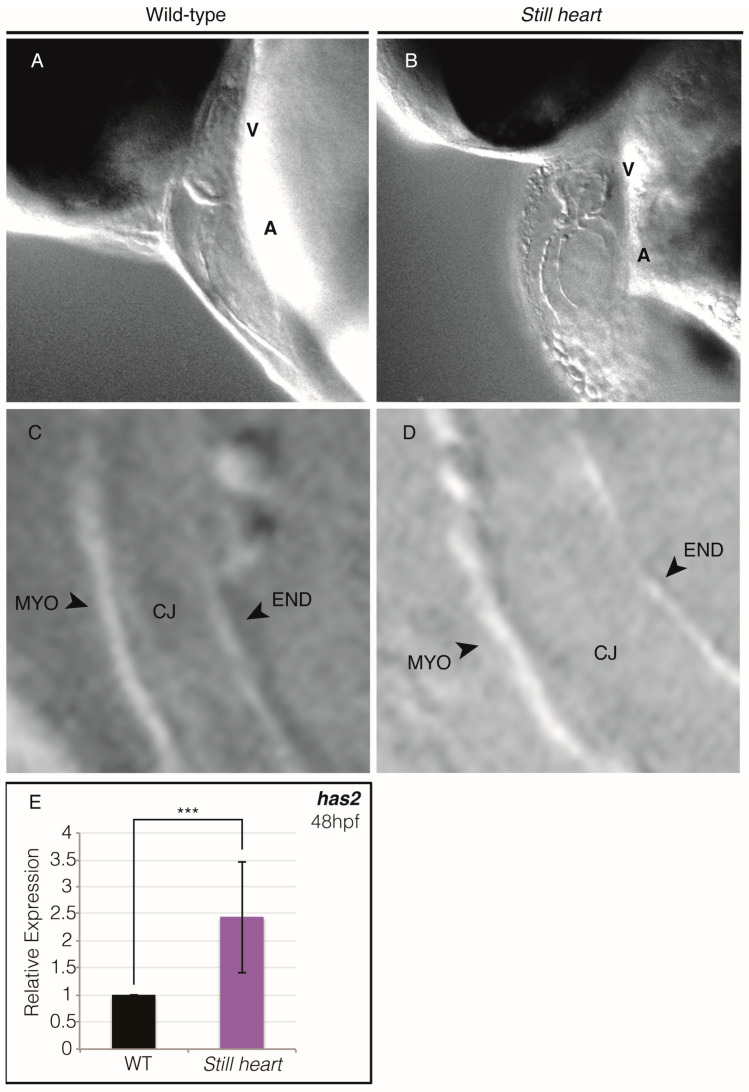
*Still heart* mutant hearts do not loop and display an expansion of cardiac jelly. At 48 hpf, wild-type hearts have looped, with the ventricle and atrium chambers in different planes of focus (**A**). Unlike wild-type, our *still heart* mutant hearts do not loop, which allows for easy visualization of both chambers in a single plane of focus at 48 hpf (**B**). Wild-type hearts (**C**) display a thin layer of cardiac jelly (CJ) between the endocardial (END) and myocardial (MYO) layers when compared to *still heart* mutant hearts (**D**). Panels C and D are magnifications of the atrial walls from panels A and B, respectively. Quantitative PCR analysis of *hyaluronan synthase 2* (*has2*) at 48 hpf showed that the relative fold change in expression of *has2* was significantly higher in *still heart* mutants when compared to wild-type embryos (**E**). *** = *p* ≤ 0.001.

*Still heart*, a zebrafish *smyd1b* mutant, is characterized by almost complete skeletal muscle paralysis and a nonfunctional heart [24,30]. At 24 hpf, *still heart* embryos appear indistinguishable from their wild-type siblings except for their nonfunctional cardiac and fast muscle tissues. As development continues, *still heart* mutant hearts fail to complete morphogenesis, remaining as a linear heart tube (Figure 1). Consequently, the *still heart* larvae develop severe cardiac and generalized edema, and the nonfunctional heart becomes taut (Supplementary Figure S1).

In addition to failing to loop, *still heart* mutant hearts display a significant separation of endo- and myocardium that is indicative of an increase in the extracellular matrix (ECM), also known as cardiac jelly, between these cardiac layers (Figure 1). The expansion of the cardiac jelly can be due to the increased production of ECM components such as hyaluronic acid by hyaluronan synthase 2 (Has2) [47]. We examined the expression of *has2* in the *still heart* mutants at 48 hpf and found a significant increase in *has2* expression (Figure 1E), suggesting that unregulated *has2* expression may be responsible for the expansion of the cardiac jelly observed in *still heart* mutants.

### 3.2. SMYD1b Is Necessary for Proper Regulation of the Cardiac Transcriptional Network

**Figure 2:**
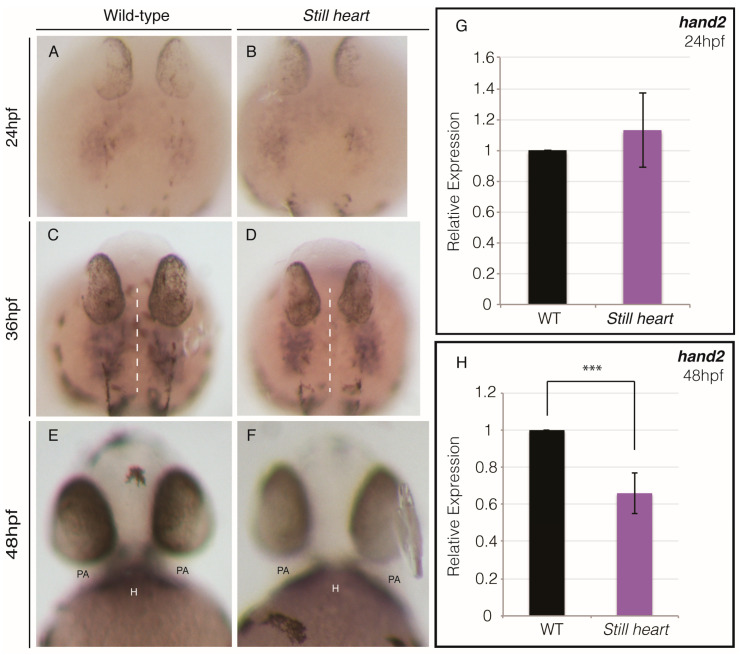
*Still heart* mutants lack *hand2*-migrating cells and *hand2* expression in their hearts. At 24 hpf, *hand2* expression can be observed in the bilateral cell populations of the anterior lateral plate mesoderm in both wild-type (**A**) and *still heart* mutants (**B**). By 36 hpf, *hand2* expression has expanded toward the midline (dotted white line) of wild-type embryos (**C**), while there is a notable void of *hand2* expression proximal to the midline in *still heart* mutants (**D**). At 48 hpf, wild-type embryos display *hand2* expression in their pharyngeal arches (PAs) and heart (H) (**E**). *Still heart* mutants demonstrate *hand2* expression in their pharyngeal arches but lack *hand2* expression in their hearts (**F**). Comparison of relative *hand2* expression in wild-type and *still heart* mutants revealed no significant difference in *hand2* expression at 24 hpf (**G**). However, by 48 hpf, *still heart* mutants demonstrate a significant reduction in the relative fold change in *hand2* expression (**H**). *** = *p* ≤ 0.001.

In mice, Smyd1 is required for *hand2* transcription during heart morphogenesis and specification of the right ventricle [22]. To test if Smyd1-dependent *hand2* expression is conserved in zebrafish heart development, we assessed the expression of *hand2* at 24, 36, and 48 hpf (Figure 2). At 24 hpf, *hand2* is expressed in the anterior lateral plate mesoderm (ALPM) with no significant difference in the level of *hand2* expression between the wild-type and *still heart* embryos (Figure 2A,B,G). At 36 hpf, *hand2*-expressing cells are observed proximal to the embryonic midline in the wild-type siblings, whereas the *still heart* embryos are void of *hand2* expression approaching the midline (Figure 2C,D). By 48 hpf, the *still heart* embryos demonstrate a significant reduction in hand2 expression and lack *hand2* expression in their hearts, although the expression in the neighboring pharyngeal arches is normal (Figure 2E,F,H). The heart-specific reduction in hand2 expression in the zebrafish *still heart* mutants is consistent with findings from *hand2* assays in mice [22].

**Figure 3:**
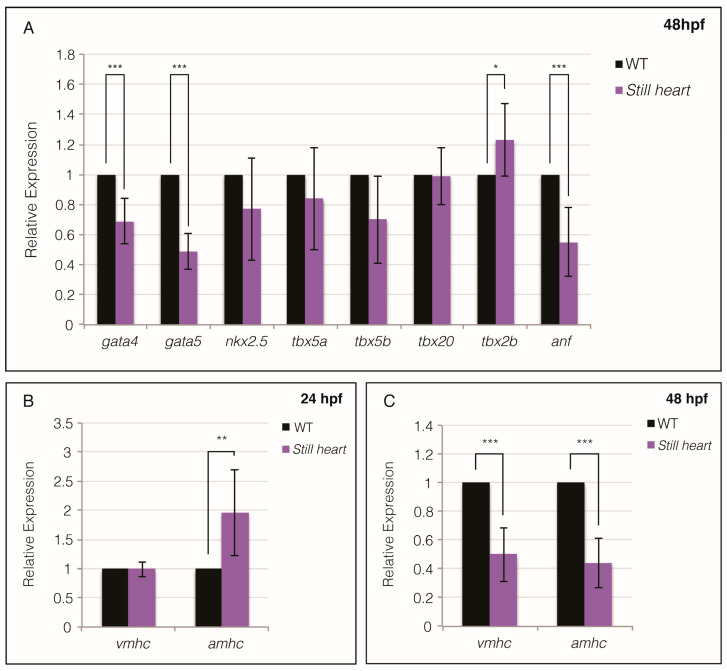
*Still heart* mutants have altered expression of key cardiac transcription factors and cardiac myosins. At 48 hpf, *still heart* mutants demonstrated a significant reduction in the relative fold change in expression of *gata4*, *gata5*, and *anf* compared to wild-type (**A**). Other key transcription factors, *tbx5a*, *tbx5b*, and *tbx20*, did not show a significant change in expression in *still heart* mutants, while the relative expression of *tbx2b* was elevated. Comparison of cardiac myosin heavy chain expression revealed no significant difference in *vmhc* expression between *still heart* and wild-type embryos at 24 hpf (**B**). *Still heart* mutants displayed a significantly higher relative fold change in *amhc* expression at 24 hpf. By 48 hpf, the relative fold change in expression of *vmhc* and *amhc* is significantly lower in *still heart* mutants when compared to wild-type (**C**). * = *p* ≤ 0.05, ** = *p* ≤ 0.01, *** = *p* ≤ 0.001.

To investigate whether Smyd1b affects the transcription of other cardiac factors, we examined the expression of core cardiac transcription factors and chamber-specific cardiac myosin heavy chains (Figure 3). The *still heart* mutants displayed reduced expression of *gata4 and gata5*, which are direct transcriptional activators of *hand2* [22]. The *nkx2.5* expression was reduced in the *still heart* mutants at 48 hpf, while *tbx5a, tbx5b*, and *tbx20* showed no significant difference (Figure 3A). *Atrial natriuretic factor* (*anf/nppa*) is a direct transcriptional target of the Gata4, Nkx2.5, and Tbx5 complexes [48] and *anf* expression is significantly reduced in the *still heart* mutants (Figure 3A), further supporting evidence of the dysregulation of one or more core cardiac transcription factors. Additionally, *tbx2b*, which represses *anf* and activates *has2* transcription [49], demonstrates increased expression in *still heart mutant* embryos (Figure 3A).

Smyd1b, a histone methyltransferase with a myosin-binding domain, is essential for the proper assembly of fast skeletal and cardiac sarcomeres, specifically regarding myosin folding and incorporation [24,30,50]. We have previously shown that fast skeletal muscle myosin expression is comparable between wild-type and *still heart* embryos at 24 hpf [24]. To assess whether cardiac sarcomere myosin expression is affected in *still heart* mutants, we examined the expression levels of *ventricular myosin heavy chain* (*vmhc*) and *atrial myosin heavy chain* (*amhc*). At 24 hpf, *still heart* mutants showed similar *vmhc* expression levels to wild-type, while *amhc* expression was significantly upregulated (Figure 3B). By 48 hpf, the qPCR analysis indicated a significant reduction in *vmhc* and *amhc* expression (Figure 3C), suggesting that there is a change in the transcriptional regulation of these chamber-specific myosin genes or that there is a reduction in the cell number or tissue size expressing these myosins.

### 3.3. Still Heart Mutant Hearts Lack Contribution from the Second Heart Field

**Figure 4:**
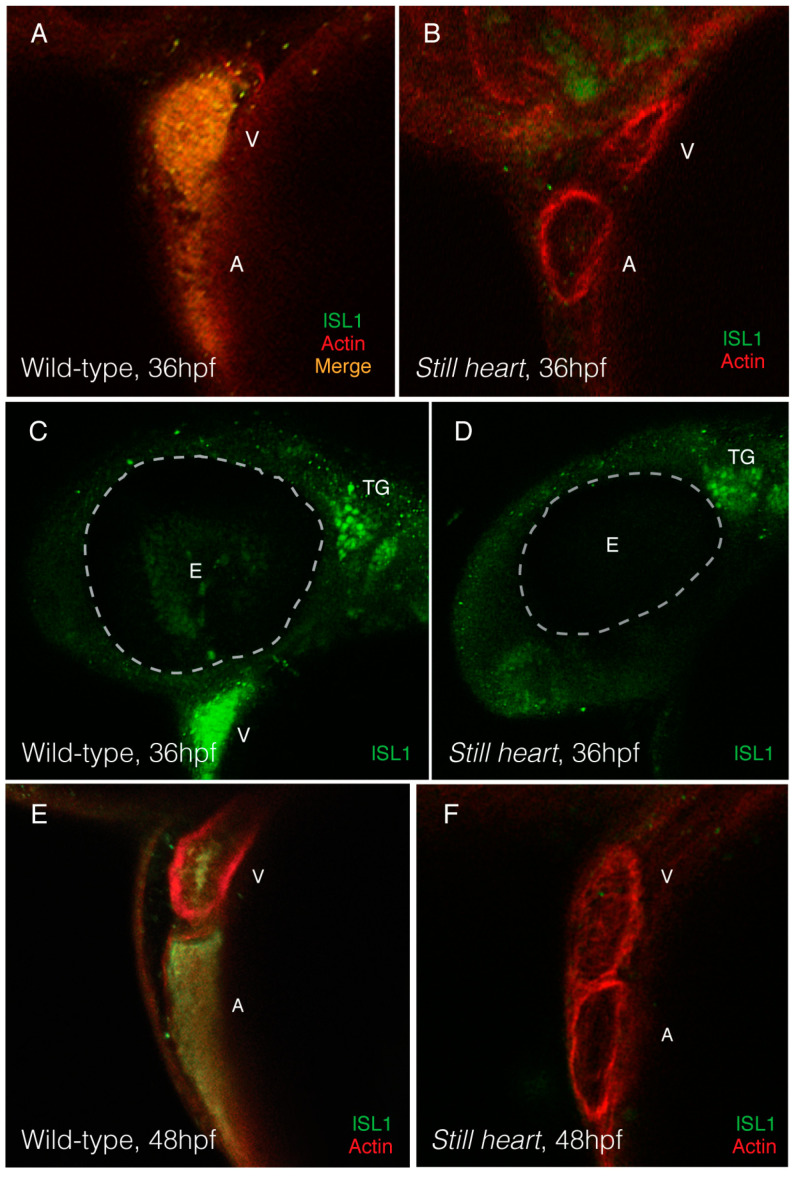
*Still heart* mutant hearts lack incorporation of Isl1-positive cells from the second heart field. At 36 hpf, immunostaining against Isl1, a marker of second heart field cardiac progenitor cells and cardiac neural crest, revealed a lack of Isl1-positive staining in the hearts of still heart mutants when compared to wild-type embryos (**A**–**D**). Although *still heart* mutant hearts did not display any Isl1 staining, other tissues derived from neuronal lineages, such as the trigeminal ganglion (TG), were positive for Isl1 (**C**,**D**). *Still heart* mutants continue to lack Isl1 staining in their hearts at 48 hpf when compared to wild-type embryos of the same age (**E**,**F**). (Ventricle = V; atrium = A; eye = E).

Many transcription factors that are misregulated in *still heart* mutants play crucial roles in the second heart field (SHF) and its contribution to heart development [5,6,11,51,52]. The SHF is a population of multipotent cells outside of the heart, adding cells to both cardiac poles throughout heart morphogenesis. This process of cell migration, differentiation, and integration requires the coordination of several factors, such as *gata4*, *nkx2.5*, *tbx5a*, *tbx5b*, *tbx20*, and *hand2*, to incorporate SHF cells into the developing heart [6,11,52,53]. Given the altered expression of multiple transcription factors in *still heart* mutants and the reduced expression of myosin heavy chains, which may suggest a smaller cardiomyocyte population size, we wanted to determine whether the addition of SHF cells to the primary heart field is affected. Islet-1 (Isl1) is a well-established marker of SHF progenitor and cardiac neural crest cells [15,40,54]. We examined the wild-type and *still heart* mutants at 36 and 48 hpf for Isl1-positive cells to determine if SHF cells make it to the heart. Isl1 was absent in the *still heart* mutant hearts at 36 and 48 hpf when compared to the wild-type embryos of the same stage (Figure 4). Although Isl1-positive cells were absent from the mutant heart, Isl1 was present in other cell populations of neural lineage, such as the trigeminal ganglion, suggesting that only the heart is affected in *still heart* mutants.

## 4. Discussion

The results presented here highlight the critical role of Smyd1b in zebrafish heart morphogenesis, and particularly the requirement of Smyd1b for the development of the second heart field. Mutations that affect the transcriptional regulation and contribution of the SHF to the heart are associated with congenital heart disease in humans and mice [8]. Laboratory-studied *smyd1* mutations are largely embryonic lethal [22,41,55] and very few *smyd1* mutations have been identified in human patients, with varying phenotypic severity [27,56,57,58,59]. We demonstrate that zebrafish mutant in *smyd1b* (*still heart*) exhibit disruptions in heart development such as a failure to loop, the excessive expansion of the cardiac extracellular matrix, the abnormal regulation of cardiac transcription factors, and an absence of SHF-derived cells in the heart.

Our data support previous findings that Smyd1b is essential for heart morphogenesis and development [22,23,24,30,41]. Unlike wild-type hearts, which develop a characteristic looped structure, *still heart* mutants display a nonfunctional linear heart tube that fails to complete looping and results in significant edema due to lack of circulation (Figure 1, Supplementary Figure S1). The observed increase in cardiac jelly—resulting from elevated *has2* expression (Figure 1E)—is likely a key contributor to the separation of the endocardium and myocardium in the *still heart* embryos. The expansion of the ECM likely interferes with heart morphogenesis, since the overproduction of ECM components leads to myocardial stiffness, as seen in myocardium following myocardial infarctions and pathological mechanical stress [60,61]. Whether *has2* is a direct transcriptional target of Smyd1b remains unknown but the expression of several cardiac factors is abnormal, including *tbx2b*, which does activate *has2* transcription [49].

**Figure 5:**
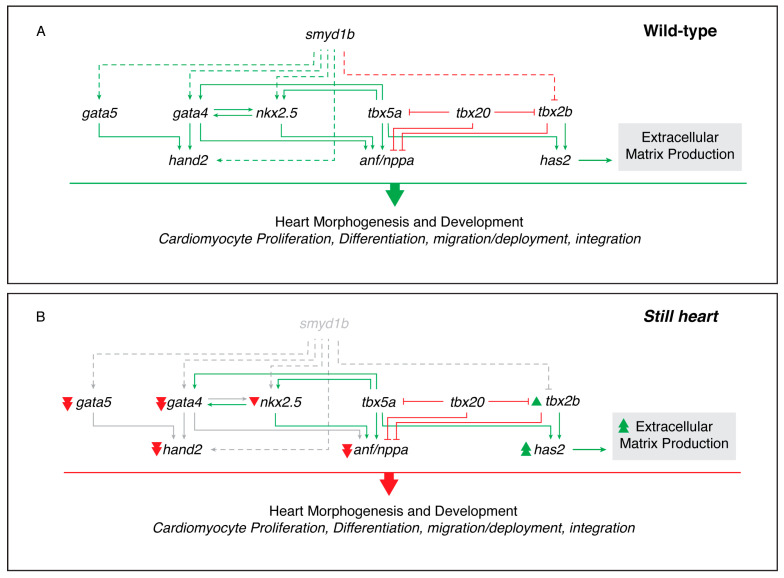
Transcriptional changes in cardiac factors in *still heart* mutants. A schematic representation of the altered expression of key cardiac transcription factors between wild-type (**A**) and *still heart* mutants (**B**). Genes such as *gata4*, *gata5*, *hand2*, and *has2* exhibit significant changes in expression in the absence of Smyd1b. (Solid green line = directly activates transcription; dashed green line = indirectly or directly activates transcription; solid red line = directly inhibits transcription; dashed red line = indirectly or directly activates transcription; gray = absent; single green arrowhead = increased expression; double green arrowhead = significantly increased expression/production; single red arrowhead = decreased expression; double red arrowhead = significantly decreased expression).

Previous work has shown that SMYD1 is required for *hand2* expression in mice [22]. Hand2 is required for the survival, proliferation, migration, and differentiation of second heart field cells [53,62]. We examined the expression of *hand2* at three stages of heart morphogenesis and found that *hand2* expression was significantly reduced in the zebrafish *still heart* mutant hearts (Figure 2). Our work demonstrated a lack of *hand2*-positive cells proximal to the midline and in the heart of the *still heart* mutant embryos at 36 and 48 hpf, respectively. The absence of cells expressing *hand2* approaching the midline in the *still heart* mutants is likely due either to a lack of SHF progenitor cell specification or migration from the anterior lateral plate mesoderm to the heart tube. Smyd1 regulates the expression of several genes, either directly or indirectly [63,64,65,66]. To investigate whether Smyd1b indirectly activates *hand2* expression, we examined the expression of transcription factors upstream of *hand2*. The expression of *gata4* and *gata5*, direct activators of *hand2* [67], was significantly reduced in the *still heart* mutants (Figure 3A). Core transcription factors, *tbx5a, tbx5b*, and *tbx20*, showed no significant change in expression, while *nkx2.5* was slightly reduced in the *still heart* mutants (Figure 3A). These factors regulate the transcription of other genes such as *anf/nppa*, which is significantly downregulated in *still heart* mutants, suggesting the cumulative activation by Gata4 and Nkx2.5 is absent and/or the repression by Tbx2b further inhibits *anf* expression [49,68]. Several of the misregulated factors, *gata4*, *gata5*, *nkx2.5*, *tbx2b*, and *anf/nppa*, are required for proper heart morphogenesis and regulating the contribution of cardiomyocytes from the SHF [5,11,52]. The mis-regulation or absence of these transcription factors are also associated with congenital heart disease, which broadens the effect and severity Smyd1 has on vertebrate heart development [69] (Figure 5).

Our analysis of the expression of cardiac myosins, *vmhc* and *amhc*, which are the downstream factors of the cardiac transcriptional network and one of the first signs of cardiomyocyte differentiation, demonstrated a quantitatively significant reduction in mRNA expression at 48 hpf (Figure 3B,C). This reduced expression at the end stages of heart morphogenesis is likely due to a decline in the cell number, either due to a lack of cell proliferation or recruitment from the second heart field. We showed, using immunostaining against Islet-1, that the *still heart* mutant hearts lack cells from the second heart field when compared to the wild-type hearts at the same stages (Figure 4). The lack of SHF cells in the zebrafish heart coincides with the lack of a right ventricle and truncated outflow tract, both SHF-derived structures, in the Smyd1 null mice [6,55]. Cells from the second heart field compose a fair portion of the mature heart and therefore, the loss of these cells to the developing heart will result in a significant reduction in the overall gene expression and organ structure [5,14,40]. Since *Smyd1* knockouts are lethal during early mammalian heart morphogenesis, studies of *Smyd1(b)* in embryonic and larval zebrafish offer insights that can be explored in mammalian systems using conditional knockout models or stem cells, where *Smyd1* is removed from the myocardium after cardiac sarcomerogenesis and heart development are complete [55,70,71].

In summary, our findings demonstrate the essential role of Smyd1b in heart morphogenesis, specifically in supporting second heart field development. *Still heart* mutants exhibit structural and functional heart abnormalities, including a lack of looping, expanded extracellular matrix, misregulated transcriptional networks, and reduced SHF progenitor contribution. The downregulation of key transcription factors such as *hand2*, *gata4*, *gata5*, and *nkx2.5* highlights Smyd1b’s regulatory influence on SHF and heart development. This work advances our understanding of Smyd1b’s role in heart development and underscores its potential importance in cardiomyocyte differentiation and heart morphogenesis [55].

## Figures and Tables

**Table 1:** qPCR primers used in this work.

Gene	Forward Primer (5′→ 3′)	Reverse Primer (5′→ 3′)
*has2*	CAAATATGAGTCGTGGGTCTCC	CATTGAACGCACCCGAAATATG
*hand2*	GCCAAAGAAGAAAGGCGAAAG	AGCTCCAATGCCCAAACA
*tbx20*	CTCACGGATATTGAGAGGGAAAG	TGTCCTTCTTCTCCCAGAGT
*tbx5a*	GGAGCCATAAGCTCACAGTATT	CCTCTTGATGCAGTGGTAGTC
*tbx5b*	ACTGTCTCTCTCGGTCTGAATA	AGTTGGGTCATGAATGCTAGG
*nkx2.5*	AGACACGTCCACTTACAACAC	CGACGGATAGTTGCATGAGTAG
*gata4*	TGTCAGACTACCACAACAACTC	GTGTCTGAATGCCCTCTTTCT
*gata5*	GACAACACTGTGGAGGAGAAA	TTTGCGTTTCCTTGTCTGAATG
*tbx2b*	GTCCCTTTCCCTTTCATCTGT	GCGGCCATGTAGGTGTAG
*amhc*	TCTGGAGCAAACCGAAAGAG	GATCCGACTCTTGCTTCTTCTT
*vmhc*	GCTGAAGAAGGAGCAGGATAC	GCCTCCCTTCATAGCGATTT

## Data Availability

The original contributions presented in the study are included in the article/supplementary material. Further inquiries can be directed to the corresponding author.

## Supplementary Materials

The following supporting information can be downloaded at https://www.mdpi.com/article/10.3390/genes16010052/s1: Figure S1: Still heart mutant hearts remain un-looped and become stretched as larvae develop generalized edema.
