# Supplementary material for: Heart Morphogenesis Requires Smyd1b for Proper Incorporation of the Second Heart Field in Zebrafish
Source: Genes (Basel). 2025 Jan 4;16(1):52. doi: 10.3390/genes16010052 (PMC11764850; doi:10.3390/genes16010052)

Supplementary Figure 1. *Still heart* mutant hearts remain un-looped and become stretched as larvae develop generalized edema. At 3 days post fertilization (dpf), wild-type hearts have defined structures and circulate blood around the body (A). *Still heart* mutant hearts have not looped and appear as two beads on a string as the heart is stretched from the surrounding pericardial edema (B). By 5 dpf, wild-type zebrafish hearts have completed morphogenesis, and the larvae appear streamlined (C), while the *still heart* mutants have pericardial (black arrowhead) and generalized edema (red arrowhead) with a nonfunctional heart that remains taut. (V = ventricle; A = atrium; BA = bulbus arteriosus).

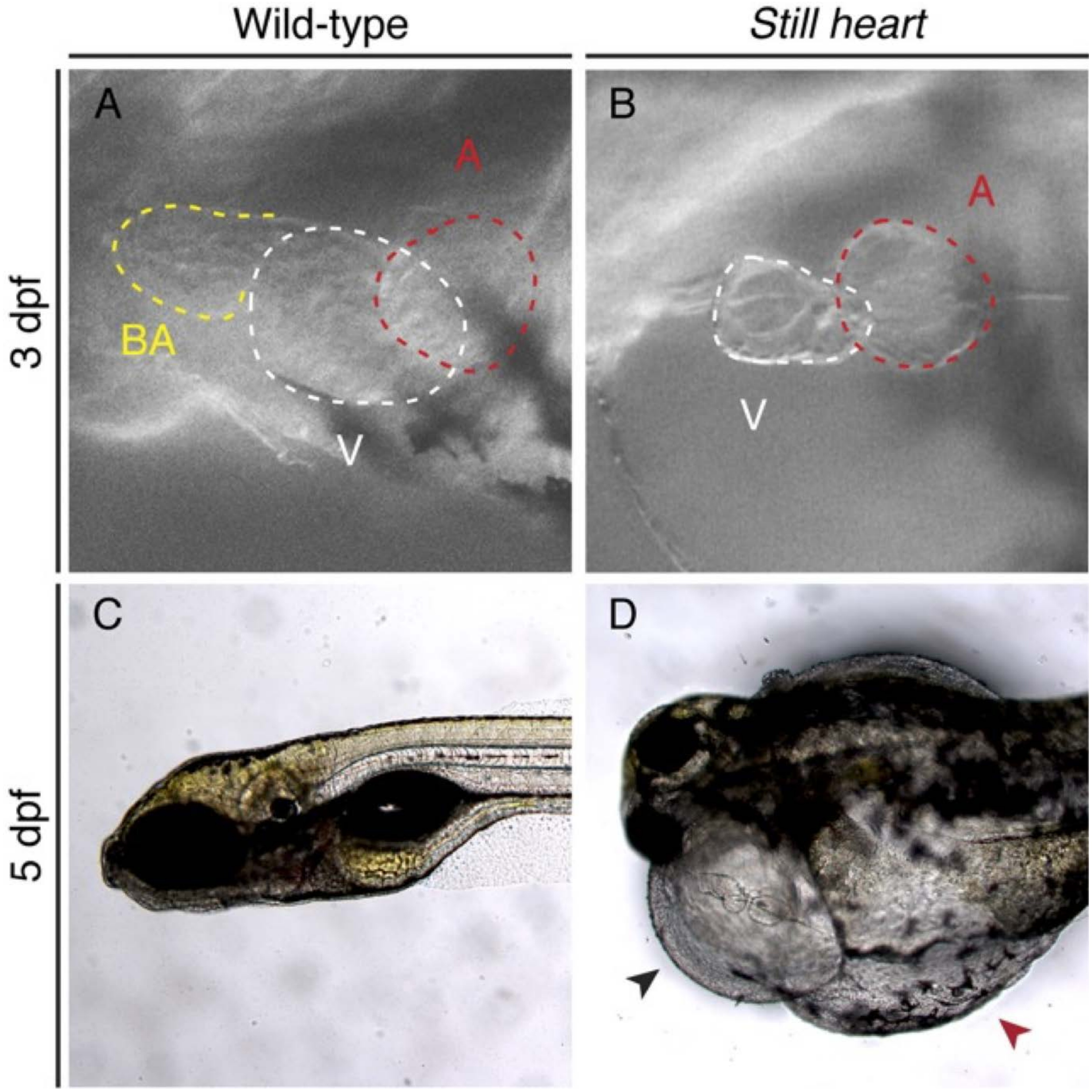

Supplement: Supplementary file 1 [file genes-16-00052-s001.zip › Supplementary Figure S1.pdf]
